# Molecular Detection and Species Determination of Malaria Parasites, Venezuela

**DOI:** 10.3201/eid2502.181279

**Published:** 2019-02

**Authors:** César Pacheco, Jorge Moreno, Flor Herrera

**Affiliations:** Author affiliations: University of Carabobo, Aragua, Venezuela (C. Pacheco, F. Herrera);; IAE Dr. Arnoldo Gabaldon, Field Research Center “Dr. Francesco Vitanza,” Tumeremo, Venezuela (J. Moreno)

**Keywords:** Plasmodium falciparum, Plasmodium vivax, Venezuela, malaria, vector-borne infections, parasites, molecular detection

## Abstract

In southeastern Venezuela, malaria cases have increased since 2013. We found that 46% of 352 blood samples from symptomatic patients in 1 municipality tested positive for *Plasmodium* spp. In addition, the number of cases increased by 10 times in 4 years (2014–2017) and by 3 times in 1 year (2016–2017).

In 1961, the Bolivarian Republic of Venezuela was the first country to be certified by the World Health Organization as malaria-free (*1*). However, because of economic and political crises since 2013, the number of malaria cases has increased alarmingly, especially in mining towns in the Sifontes municipality, Bolivar State (*2*). The estimated number of malaria cases reported by surveillance systems in 2017 was higher than the annual average documented during the previous 29 years (1988–2016) (*3*). *Plasmodium falciparum* and *P. vivax* are the most important species of *Plasmodium* in Venezuela. *P. falciparum* causes the most severe malaria and can develop multiresistance to conventionally used antimalarial drugs (*4*). We aimed to assess the number of malaria cases, distributed by species, in symptomatic patients at a public health center in Sifontes municipality during epidemiologic week 50, 2016, and compare them with cases from the same week during 2014, 2015, and 2017.

## The Study

**Figure 1:**
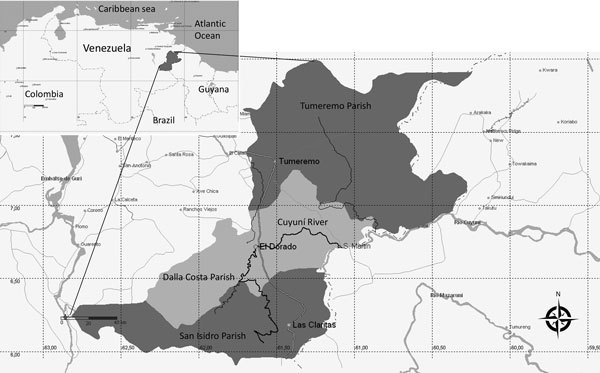
Geographic area of the Sifontes municipality, Bolivar state, Venezuela, with its parishes. Inset shows location of Bolivar state within Venezuela and proximity to other countries.

We conducted our study in Sifontes municipality (6°00′–7°54′N, 60°44′–61°39′W), Bolivar State, Venezuela (Figure 1). This municipality is a meso-endemic focus of unstable malaria, and transmission occurs during the entire year; outbreaks are associated with gold mining activities (*5*). Sifontes is divided into 3 parishes: Tumeremo in the north, El Dorado in the middle, and San Isidro in the south. San Isidro Parish has one of the largest deposits of gold in the country (Las Cristinas mine) and represents the focus with the highest malaria incidence during the last decade (*5*).

Blood was collected by ear prick from 352 patients (247 male, 105 female) who had malaria symptoms (high fever, profuse sweating, headache, nausea, abdominal pain, diarrhea) after they provided informed consent approved by the Bioethics Committee of the Biomedical Research Institute of the University of Carabobo (BIOMED-UC). Most (≈90%) patients came from San Isidro parish to be treated at the Field Research Center “Dr. Francesco Vitanza,” a public health center in the city of Tumeremo, during epidemiologic week 50 (December 12–17), 2016. First, blood samples (≈3 drops) were collected directly on microscope slides and then a similar amount of blood was collected on filter paper. Microscopic diagnosis of malaria was performed promptly; filter papers were air dried, stored in vials, transported at 4°C to BIOMED-UC, and stored at −20°C until processing. 

The principal method for malaria detection has been microscopic and in Venezuela is performed as part of a regular procedure for diagnosing malaria (*6*). However, previous studies have found that PCR typing has higher sensitivity (97.2%) and specificity (100.0%) than microscopy (*7*); therefore, we used PCR to detect parasitic infections. We extracted DNA from individual samples using the phenol/chloroform method, resuspended in 50 μL sterilized water, and stored at −80°C (*8*). We detected and typed *Plasmodium* spp. in blood samples using a previously described assay (*9*). This nested PCR uses *Plasmodium* genus-specific primers rPLU6 (5′-TTAAAATTGTTGCAGTTAAAACG-3′) and rPLU5 (5′-CCTGTTGTTGCCTTAAACTTC-3′) for the initial PCR amplification, followed by species-specific primers for the second amplification: *P. falciparum* rFAL1 (5′-TTAAACTGGTTT GGGAAAACCAAATATATT-3′) and rFAL2 (5′-ACACAATGAACTCAATCATGACT ACCCGTC-3′) and *P. vivax* rVIV1 (5′-CGCTTCTAGCTTAATCCA CATAACTGATAC-3′) and rVIV2 (5′- ACTTCCAAGCCGAAGCAAAGAAAGTCC TTA-3′). The PCR amplifications were conducted in 25-μL reaction volumes using 50 ng of template DNA in a PTC-100 thermal cycler (MJ Research, Inc., http://mj-research.com). Negative controls (all reagents except template) were run to detect possible contamination. The amplified products were visualized by electrophoresis in 2% agarose gel stained with syber green. We estimated the percentage of parasite species in the detected cases was estimated as the percentage of positive *Plasmodium* samples divided by the total number of samples assayed.

**Figure 2:**
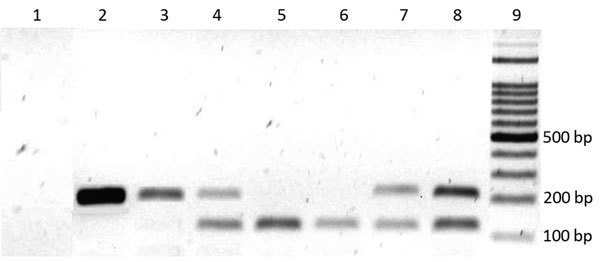
Detection of *Plasmodium* spp. in blood samples by gel electrophoresis on a 2% agarose gel, Venezuela. DNA amplicons generated by nested PCR of the DNA extracted from malaria parasites in blood samples from patients. Lane 1, negative control; lanes 2 and 3, *P. falciparum*–infected samples; lanes 4 and 7, mixed infection (*P. falciparum* + *P*. *vivax*) samples; lanes 5 and 6, *P. vivax*–infected samples; lane 8, mixed positive controls of *P. falciparum* (205 bp) and *P. vivax* (120 bp); lane 9, 100-bp ladder marker.

Microscopy and PCR revealed the presence of *P. falciparum* and *P. vivax* in blood samples from patients with suspected malaria infections. However, microscopy failed to detect malaria parasites in 2 samples in which PCR detected it, and 1 infection determined to be mixed by PCR was detected as monoinfection by microscopy. We can state therefore that microscopy and PCR yielded similar results. Figure 2 shows the specific size DNA amplicons of each species seen in the agarose gel. The negative control was consistently negative in all PCR runs. The malaria-positive rate by PCR was 46.0% (162/352). Among these 162 positive samples, 124 (76.5%) were detected in men and 38 (23.5%) in women; 115 (92.7%) of 124 infected men worked in mines. *P. vivax* accounted for 101 (62.3%) infections, *P. falciparum* for 50 (30.9%) infections, and mixed species for 11 (6.8%) infections.

Malaria infections and their breakdown by species, detected in the same center by microscopy in the same week 50 in other years, were as follows: 2014, 50 cases, 76% *P. vivax* and 24% *P. falciparum*; 2015, 64 cases, 95.3% *P. vivax* and 4.7% *P. falciparum*; 2017, 503 cases, 68.4% *P. vivax*, 28.4% *P. falciparum*, and 3.2% mixed infections. The number of cases during these 4 years in this public health center averaged 14% of all cases in Sifontes municipality.

The Venezuela Ministry of Health has not published epidemiologic data since November 2015. Therefore, we obtained comparison data on the number of positive cases directly from the healthcare center staff. In other states in Venezuela, malaria is also increasing. Reports from the Sucre State Department of Health indicated a 126-fold increase of *P. falciparum* malaria from 2011 to 2015 (*10*). Persons from Venezuela and other countries, attracted to mining activities, are moving to Bolivar state to work there for periods of time and then return home, especially when sick (*11*). Therefore, malaria could spread from the Sifontes municipality to other regions through the action of malaria vectors. 

## Conclusions

The rate of malaria infection (46%) we found could represent the current malaria prevalence among symptomatic patients (115 [71%] men of 162 malaria patients worked in mines) attending the public health center during week 50 in Bolivar state. The results also show that the number of positive cases increased by 10 times in 4 years (2014–2017) and by 3 times in only 1 year (from 2016 to 2017). Therefore, the tendency of malaria cases to increase is very high. With respect to the *P. falciparum* infections, results suggest that this species is increasing with time except for 2015, when infection rates were low.

Several reasons could explain the increase in malaria prevalence in the public health center during epidemiologic week 50 from 2014 to 2017: 1) insufficient antimalarial drugs, which creates irregular distribution and unsupervised administration of them (*12*); 2) resistance to antimalarial drugs (*4*); and 3) increases in mosquito breeding habitats and rise in secondary vectors from malaria transmission attributable to deforestation by mining activities (*13*). Therefore, Venezuela, as well as neighboring countries that receive malaria patients from Venezuela, should strengthen vector-control efforts and improve malaria diagnosis and treatment.
